# Aconitase 2 inhibits the proliferation of MCF-7 cells promoting mitochondrial oxidative metabolism and ROS/FoxO1-mediated autophagic response

**DOI:** 10.1038/s41416-019-0641-0

**Published:** 2019-12-10

**Authors:** Fabio Ciccarone, Luca Di Leo, Giacomo Lazzarino, Giuseppe Maulucci, Flavio Di Giacinto, Barbara Tavazzi, Maria Rosa Ciriolo

**Affiliations:** 1 Department of Biology, University of Rome “Tor Vergata”, Via della Ricerca Scientifica 1, Rome, 00133 Italy; 2 UniCamillus-Saint Camillus International University of Health Sciences, via di Sant’Alessandro 8, 00131 Rome, Italy; 3 Fondazione Policlinico Universitario A. Gemelli IRCCS, Largo A. Gemelli 8, 00168 Rome, Italy; 4 Institute of Physics, Catholic University of Rome, Largo F. Vito 1, 00168 Rome, Italy; 5 Institute of Biochemistry and Clinical Biochemistry, Catholic University of Rome, Largo F. Vito 1, 00168 Rome, Italy; 6 IRCCS San Raffaele Pisana, Via della Pisana 235, Rome, 00163 Italy; 7 Present Address: Danish Cancer Society Research Center, Unit of Cell Stress and Survival, Strandboulevarden 49, DK-2100 Copenhagen, Denmark

**Keywords:** Cancer metabolism, Autophagy

## Abstract

**Background:**

Deregulation of the tricarboxylic acid cycle (TCA) due to mutations in specific enzymes or defective aerobic metabolism is associated with tumour growth. Aconitase 2 (ACO2) participates in the TCA cycle by converting citrate to isocitrate, but no evident demonstrations of its involvement in cancer metabolism have been provided so far.

**Methods:**

Biochemical assays coupled with molecular biology, in silico, and cellular tools were applied to circumstantiate the impact of ACO2 in the breast cancer cell line MCF-7 metabolism. Fluorescence lifetime imaging microscopy (FLIM) of NADH was used to corroborate the changes in bioenergetics.

**Results:**

We showed that ACO2 levels are decreased in breast cancer cell lines and human tumour biopsies. We generated ACO2- overexpressing MCF-7 cells and employed comparative analyses to identify metabolic adaptations. We found that increased ACO2 expression impairs cell proliferation and commits cells to redirect pyruvate to mitochondria, which weakens Warburg-like bioenergetic features. We also demonstrated that the enhancement of oxidative metabolism was supported by mitochondrial biogenesis and FoxO1-mediated autophagy/mitophagy that sustains the increased ROS burst.

**Conclusions:**

This work identifies ACO2 as a relevant gene in cancer metabolic rewiring of MCF-7 cells, promoting a different utilisation of pyruvate and revealing the potential metabolic vulnerability of ACO2-associated malignancies.

## Background

The TCA cycle represents the core pathway for aerobic oxidation of carbohydrates, lipids and proteins in mitochondria, by supplying the reduced coenzymes NADH and FADH_2_ necessary for ATP production through the oxidative phosphorylation (OXPHOS).^1,2^ However, many cancer cells prefer to enhance the glycolytic rate for energetic purposes, by favouring glucose transporters and glycolytic enzymes rather than the TCA cycle machinery, and this results in increased lactate production even in normoxia, a phenomenon known as aerobic glycolysis or the ‘Warburg effect’.^3^ This peculiarity is significant as the intermediates of the TCA cycle can diverge towards anabolic reactions, leading to amino acids, lipids and nucleotide synthesis necessary for supporting the high rate of proliferation.^1,2^ Based on this, the manipulation of glycolysis and of the TCA cycle reactions by cancer cells represents a core strategy for their metabolic demands, such as energy production, biomass assimilation and redox control. In this effort, many cancer cells avidly catabolise several metabolites among which is glutamine that, by providing alfa-ketoglutarate (α-KG), replenishes the anabolic reactions of the TCA cycle for lipid and nucleotide synthesis, as well as for redox homoeostasis and protein O-GlcNAcylation.^4,5^ Other well-established metabolites used to sustain cancer growth and adaptation include, for instance, acetate and fatty acids, and this metabolic plasticity expands the concept beyond the oncogene-driven metabolic rewiring.^3,6,7^ The classical idea that the establishment of the Warburg effect is solely a consequence of dysfunctional mitochondria, as suggested by initial associations with mutations affecting TCA cycle or electron transport chain (ETC) proteins, is completely changed in a more realistic view of a cancer cell that constantly readapts to tumour microenvironment fluctuations, including nutrient and oxygen availability.^1,3^ In fact, many studies have clearly demonstrated that cancer cells relying on aerobic glycolysis retain intact mitochondrial pathways,^8,9^ including biogenesis and mitophagy that greatly improve metabolic networks in response to nutrient availability. Moreover, the enhancement of oxidative capacities by reorientation of metabolites towards mitochondria^10,11^ or by the disposal of damaged mitochondria via mitophagy^12^ was demonstrated to be harmful to cancer cells addicted to the Warburg effect. Consistently, interfering with tumour metabolic phenotype is a novel therapeutic strategy that can be exploited to preferentially kill cancer cells.^13^

Considering the advantages, in terms of energetic and anabolic precursors, that a cancer cell can acquire by deregulating the TCA cycle, in this paper, we have assessed whether the modulation of aconitase 2 (ACO2), the second enzyme involved in the TCA cycle, could induce metabolic rearrangements and proliferation defects in cancer cells. This enzyme is also known as mitochondrial aconitase as it catalyses the reversible isomerisation of citrate to isocitrate in mitochondria, a reaction that can be also performed in the cytosol by aconitase 1 (ACO1). We have focused the attention on ACO2 because it belongs to a branch of the TCA cycle particularly important for cancer metabolic features as it is interposed between citrate, which plays a role in lipid anabolism, and α-KG that can be replenished by glutamine anaplerosis.^1,2^ Moreover, other relevant cues in support of a putative involvement of ACO2 in cancer are that (i) its reduced levels in pluripotent stem cells make glutamine a key fuel for the TCA cycle,^14^ (ii) it was found inactivated in fumarate hydratase (FH)-deficient tumours^15^ and (iii) its expression was found downregulated in cancer cells.^16,17^

Here we show that ACO2 expression is reduced in breast cancer, and increasing the levels of the enzyme in MCF-7 cells can inhibit cell proliferation. By generating cells with stable overexpression of *ACO2* gene, we demonstrated that proliferation inhibition is associated with enhanced oxidative metabolism mainly due to redirection of pyruvate to mitochondrial oxidation. The metabolic phenotype of ACO2-expressing cell was favoured by autophagic/mitophagic response to increased production of reactive oxygen species (ROS) by the activation of Forkhead box protein O1 (FoxO1) signalling.

## Methods

### Cell cultures, transfections and treatments

Cell lines were grown in Dulbecco’s modified Eagle’s medium (DMEM) (Lonza) supplemented with 10% foetal bovine serum (EuroClone), 10 U/ml penicillin/streptomycin (Lonza) and 2 mM L glutamine (Lonza). Cells were authenticated and characterised by the supplier. Mycoplasma test was routinely carried out according to protocols from our laboratory. Cells were cultured at 37 °C in an atmosphere of 5% CO_2_ in air. For transfection of ACO2 in MCF-7, the pCMV6-Entry-ACO2 plasmid (kindly provided by Prof. Kamp DW, Jesse Brown VA Medical Center and Northwestern University Feinberg School of Medicine, Chicago) was used and selection obtained by 500 µg/ml G418 (Sigma-Aldrich). Cells were treated with 20 µM Bis-2-(5-phenylacetamido-1,3,4-thiadiazol-2-yl)ethyl sulfide (BPTES) (Sigma-Aldrich) for 24 h, 5 mM N-acetylcysteine (NAC) (Sigma-Aldrich) for 24 h, 30 µM chloroquine (CQ) (Sigma-Aldrich) for 2 h, 50 nM Bafilomycin A1 (BafA1) (Sigma-Aldrich) for 24 h, 5 µM MitoTEMPO (Sigma-Aldrich) for 24 h and 10 mM L-ascorbic acid (Sigma-Aldrich) for 24 h. For galactose treatment, cells were incubated for 48 h with glucose-free medium containing 10 mM galactose or 10 mM glucose.

### Western blot analyses, mitochondrial and nuclear fractionation

Protein lysates were obtained by incubation of total, nuclear and mitochondrial fractions on ice for 20 min in lysis buffer (50 mM Tris-HCl, pH 7.4, 150 mM NaCl, 1 mM EDTA, 1% Triton X-100, 0.5% sodium deoxycholate, 0.1% SDS, 10 mM NaF, 5 mM Na_4_P_2_O_7_ and 2 mM Na_3_VO_4_) supplemented with protease inhibitor cocktail (AMRESCO) and followed by sonication. Lowry’s method was used for protein concentration before performing electrophoresis by SDS-PAGE and blotting onto a nitrocellulose membrane (Bio-Rad). The following primary antibodies were used: ACO2 (Novus Biologicals), IRP1/ACO1, GRP75, p-CREB, CREB, LAMIN B1, TOM20, HSP60, HSP75/TRAP1, AMPK, p-AMPK (Santa Cruz Biotechnology), IDH2, NRF1, PDHB (Abnova), β-ACTIN, PARKIN, FoxO1, p-FoxO1, ACTININ, ALBUMIN, DRP1, p-p70S6K, mTOR, p-mTOR, PARP-1 (Cell Signaling Technology), PGC-1α (Calbiochem), H3, UBIQUITIN (Merck Millipore), α-tubulin, LC3B (Sigma-Aldrich), FH (GeneTex) and total OXPHOS Human WB Antibody Cocktail (Abcam). Nuclear extraction was performed as previously described in Ciccarone et al.^18^ Mitochondrial purification was obtained by resuspending cells in mitochondria isolation buffer (MIB) composed of 1 mM EGTA, pH 7.4, 5 mM MOPS, 5 mM KH_2_PO_4_, 0.1% BSA and 0.3 M sucrose. Cells were broken mechanically with dounce (10 strokes) and with a tight pestle (30 strokes) in ice. The homogenate was centrifuged at 2600×*g* for 5 min at 4 °C, and the supernatant containing mitochondria was collected while the pellet was processed as before three times to enrich mitochondrial fraction. Supernatants were then centrifuged at 15,000×*g* for 10 min at 4 °C. The pellet was washed three times with MIB and then centrifuged at 15,000×*g* for 10 min at 4 °C.

### Quantitative real-time PCR (RT-qPCR)

RNA extraction was obtained by using TRItidy G (PanReact AppliChem) according to the manufacturer’s instructions. Synthesis of cDNA was obtained from 1 µg of total RNA by using PrimeScript™ RT Reagent Kit (Perfect Real Time) (Takara), and RT-qPCR reaction was performed by using the SYBR® Premix Ex Taq (Tli RNase H Plus) (Takara) on a StepOne real-time PCR System (Applied Biosystems). All reactions were run as triplicates, and relative quantification obtained by the comparative cycle threshold method by using *ACTB* for normalisation. Primers are listed in Supplementary Table 1.

### Cell proliferation assays

Cell proliferation was evaluated by Trypan blue exclusion test procedure and by bromodeoxyuridine (BrdU) incorporation assay. For the latter, cells were incubated with 10 µM BrdU for 4 h and then fixed for 30 min in ethanol:acetic acid:water solution (18:1:1). DNA denaturation was obtained by incubation on ice for 10 min with 1 N HCl and then 10 min with 2 N HCl. Treatments with PBS/0.4% Triton X-100 solution for 10 min and then PBS/3% BSA for 1 h were performed before incubation for 16–24 h with an anti-BrdU antibody (Santa Cruz Biotechnology) followed by 1 h of incubation with an Alexa Fluor™ 568 donkey secondary antibody; nuclei were stained with 1 µg/ml of Hoechst 33342 for 5 min. Images of cells were obtained with a Delta Vision Restoration Microscopy System (Applied Precision, Issaquah, WA) equipped with an Olympus IX70 fluorescence microscope (Olympus Italia, Segrate, Milano, Italy).

### Fluorescence lifetime microscopy (FLIM) of NAD(P)H autofluorescence

FLIM data were acquired with a Nikon A1-MP confocal microscope equipped with a 2-photon Ti:Sapphire laser (Mai Tai, Spectra Physics, Newport Beach, CA) by producing 80-fs pulses at a repetition rate of 80 MHz. A PML-SPEC 16 GaAsP (B&H, Germany) multi-wavelength detector coupled to a SPC-830 TCSPC/FLIM device (B&H, Germany) was used to collect the decay data. A 60 × oil-immersion objective, 1.2 NA, was used for all experiments. Samples were excited at 750 nm. Signals were integrated into the wavelength region of 408–496 nm. For image acquisition, the pixel frame size was set to 512 × 512 and the pixel dwell time was 60 µs. The average laser power at the sample was maintained at the milliwatt level. In the FLIM images, mitochondrial NAD(P)H responses were separated by the rest of the autofluorescence (cytoplasm, nuclei) by means of fluorescence intensity analysis.^19^

### Colorimetric assays

The aconitase 2 activity was determined by using the Aconitase Activity Assay Kit (Sigma-Aldrich) following the manufacturer’s instructions. Citrate Assay Kit (Sigma-Aldrich), α-Ketoglutarate Assay Kit (Sigma-Aldrich) and Fumarate Assay Kit (Abnova) were used to measure the levels of citrate, α-ketoglutarate and fumarate, respectively. Values were normalised on total protein amount.

### Citrate synthase activity

Citrate synthase activity was determined spectrophotometrically according to Oexle et al.^20^ About 2 × 10^6^ cells were harvested and washed in ice-cold PBS. Pellets were lysed in 100 mM Tris-HCl, pH 8.1 and 0.25% Triton X-100 supplemented with protease inhibitor cocktail (AMRESCO) for 30 min in ice. A total of 25 µg of proteins were used for each enzymatic reaction performed in 250 µl of reaction buffer (100 mM Tris-HCl, pH 8.1, 0.25% Triton X-100, 0.1 5,5-dithiobis(2-nitrobenzoate) (DTNB), 0.5 mM oxaloacetate and 0.31 mM acetyl-CoA). The principle of the assay is based on the reaction between DTNB and CoA-SH to form TNB with a maximum absorbance at 412 nm. The citrate synthase activity is proportional to the intensity of the absorbance. After a delay of 5 s, the reaction proceeds at 37 °C for a period of 4 min and absorbance recorded at 10-s intervals by an Eppendorf BioSpectrometer®. Enzyme activity was represented as a change in absorbance per minute (U), normalised on total protein amount.

### Oxygen consumption and ATP measurement

Oxygen consumption was determined by using a Clark-type oxygen electrode as described in Di Leo et al.^11^ ATP levels were determined by the ATP Bioluminescence Assay Kit CLS II (Roche Applied Science) according to the manufacturer’s instructions. Values were normalised on the protein amount.

### Extracellular lactate assay

The level of extracellular lactate was measured as previously described with minor modifications.^11^ Following 24 h from cell plating, the medium was replaced with a fresh one, and after 3 h, 500 µl of cell medium was precipitated with 250 µl of 30% trichloroacetic acid. Media were frozen after centrifugation at 14,000×*g* for 20 min at 4 °C, 10 µl of supernatant was incubated for 30 min at 37 °C in 290 µl of a buffer containing 0.2 M glycine/hydrazine buffer, pH 9.2, 0.6 mg/ml NAD + and 17 U/ml LDH. NADH formation was followed at 340 nm by using an Eppendorf BioSpectrometer®.

### Cytofluorimetric analysis

Thirty minutes before the end of the experimental time, cells were incubated with 2-NBDG (100 µM) for measurement of glucose uptake, MitoTracker Green (200 nM) or Nonyl Acridine Orange (NAO) (50 nM), MitoTracker Red CMXRos (200 nM) for mitochondrial membrane potential, dihydroethidium (DHE) (50 µM) and MitoSOX Red (5 µM) for ROS determination and Bodipy 493/503 (1 µM). Cells were washed and then collected in PBS, and the fluorescence intensity immediately analysed by means of a FACScalibur instrument. In total, 10,000 events were counted, and mean fluorescence intensity expressed as arbitrary units.

### Metabolite determination by HPLC

Cell cultures were collected after 24 h from plating, washed with ice-cold PBS and subsequently centrifuged at 1890×*g* for 10 min at 4 °C. Cell pellets were deproteinised as described in detail elsewhere.^21^ Briefly, cell pellets were treated with a precipitating solution composed of CH_3_CN 75% and KH_2_PO_4_ 25% (10 mM) at pH 7.4 and then centrifuged at 20,690×*g* for 15 min at 4 °C. Supernatants were collected and subjected to two chloroform washings in order to obtain an upper aqueous phase that was directly injected onto the HPLC, and analysed to determine concentrations of uridine diphosphate (UDP), uridine-diphosphate galactose (UDP-Gal), cytosine, uridine-diphosphate-n-acetylgalactosamine (UDP-GalNAc), uridine-diphosphate-N-acetylglucosamine (UDP-GlcNAc), glutamate (Glu), glutamine (Gln) and taurine (Tau). Amino acids were analysed as ortophtalaldehyde (OPA) derivatives by using a method with precolumn derivatisation,^22^ whilst cytosine and UDP derivatives were separated and quantified according to an ion-pairing HPLC method previously set up.^21^ For both analyses, the HPLC apparatus consisted of a SpectraSystem P4000 pump and a highly sensitive UV6000LP diode array detector (ThermoElectron Italia), equipped with a 5-cm light-path flow cell, set up between 200- and 400-nm wavelength for acquisition of chromatographic runs. Data were acquired and analysed by Chrom-Quest® software package provided by the HPLC manufacturer. Separation of the various compounds was carried out by using a Hypersil 250 3 4.6-mm, 5 mM particle-size column, which was provided with its own guard column (ThermoElectron Italia). Species identification and quantification in deproteinised cell extracts were determined by matching retention times, peak areas and absorption spectra of those of freshly prepared ultrapure standards. If needed, co-chromatograms were performed by adding proper standards with known concentration to the medium samples. Concentrations of UDP, UDP-Gal, cytosine, UDP-GalNAc and UDP-GlcNAc were determined at 260-nm wavelength and those of NO_2_ and NO_3_ were calculated at 206-nm wavelength. Differently, concentrations of OPA-derivatised amino acids were calculated at 338-nm wavelength. Intracellular glutathione levels were determined as previously described.^23^

### Determination of protein carbonylation

Carbonylated proteins were detected by the OxyBlot Kit (Millipore, S7150) as previously described,^24^ by using 15 μg of total proteins that were resolved in 12% SDS-polyacrylamide gels.

### Chromatin immunoprecipitation (ChIP) analysis

ChIP assays were performed on nuclear lysates as previously described.^25^ Briefly, cells were cross-linked with 1% formaldehyde for 10 min at room temperature of 37%, and the reaction was quenched by 5 min of incubation in 0.125 M glycine. Cell monolayer was harvested in ice-cold PBS containing protease inhibitors, and nuclei isolation performed as previously described.^18^ Chromatin sonication was achieved by using Bioruptor NextGen (Diagenode) to high power. Sonicated DNA of ~500–1000 bp was pre-cleared with Protein A-coupled Sepharose beads pre-saturated with Salmon Sperm DNA and then immunoprecipitated with anti-FoxO1 antibody (Cell Signaling Technology) or normal rabbit IgGs (Santa Cruz Biotechnology) for 16 h at 4 °C. Immunoprecipitated DNA amplification was performed by using SYBR® Premix Ex Taq (Tli RNase H Plus) (Takara) on a StepOne real-time PCR System (Applied Biosystems). All reactions were run as triplicates. The results are expressed as fold enrichment with respect to IgG control. Primers used are listed in Supplementary Table 2.

### Bioinformatic analyses

ACO2 expression was assessed by Gene Expression Omnibus (GEO, http://www.ncbi.nlm.nih.gov/geo) with accession numbers GSE15852 (*n* = 43 breast tumours vs normal counterpart) and GSE294318 (*n* = 54 breast tumours vs *n* = 12 healthy breast samples) through an Affymetrix Human Genome Array. Relapse-free survival (RFS) analysis referred to breast cancer was obtained from the Pan-cancer RNA-seq present in the Kaplan–Meier Plotter database. Mutational analysis of the *ACO2* gene was performed by consulting the TGCA database.

### Statistical analysis

The results are shown as means ± SEM and derive from at least three independent experiments. Statistical evaluation was conducted by using the unpaired two-tailed Student's *t* test for metabolic analysis and enzymatic activity assays, paired Student's *t* test for cytofluorimetric analysis and RT-qPCR analysis and one-way ANOVA with post hoc Tukey test for cell counts after treatments.

## Results

### ACO2 expression is reduced in breast cancer and increasing its levels dampens cell proliferation of MCF-7 cells

**Fig. 1:**
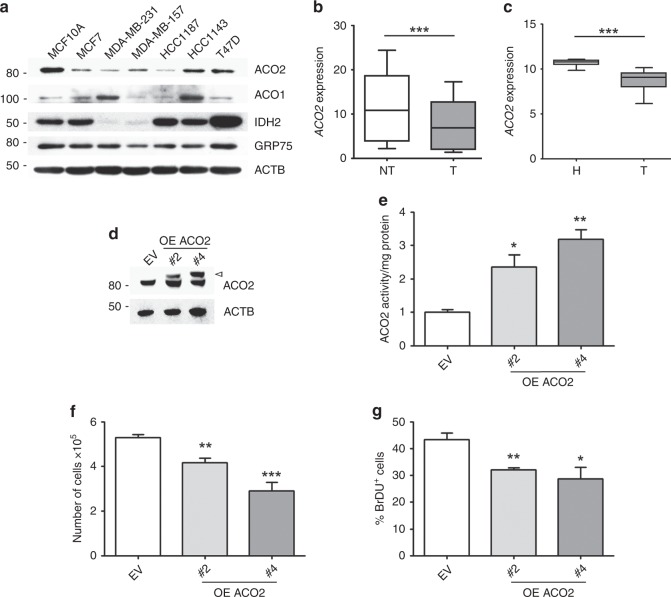
ACO2 is downregulated in breast cancer and its overexpression inhibits MCF-7 cell proliferation. **a** Representative western blot (*n* = 3) performed on breast cancer cell lines with respect to normal breast epithelial cell line MCF10A. β-actin (ACTB) was used as a loading control. **b** ACO2 expression levels in tumoural (T) breast tissue and non-tumoural adjacent counterpart (NT) obtained from GSE15852. **c** ACO2 expression levels in breast tumour biopsies (T) and normal breast biopsies form healthy subjects (H) obtained from GSE29431. **d** Representative western blot (*n* = 5) showing MCF-7 with stable overexpression of myc-ACO2 in clone 2 (#2) and clone 4 (#4) with respect to empty vector (EV)-bearing clone as a control. Arrowhead indicates the overexpressed myc-ACO2 protein. ACTB was used as a loading control. **e** ACO2 activity determination obtained by colorimetric assay (*n* = 3; *p < 0.05, **p < 0.01 vs EV). **f** Cell proliferation assayed by Trypan blue direct cell-counting procedure (*n* = 3; **p < 0.01, ***p < 0.001 vs EV). **g** Cell proliferation assayed by BrdU incorporation assay (*n* = 3; *p < 0.05, **p < 0.01 vs EV). Representative pictures of BrdU incorporation are shown in Supplementary Fig. 1h

To assess the expression of ACO2 in breast cancer cells, we compared the non-tumorigenic human breast epithelial cell line MCF10A with a panel of breast cancer cell lines, most of which exhibit a dramatic reduction of ACO2 protein levels (Fig. 1a). The decreasing trend observed for ACO2 in these cell lines was not a common feature for all the aconitase isoforms or TCA cycle enzymes, as stated by the levels of ACO1 and IDH2 proteins, respectively. Moreover, such changes were not ascribed to relevant fluctuations in mitochondrial mass, as demonstrated by the expression of GRP75 (Fig. 1a). RT-qPCR demonstrated that the transcriptional downregulation of *ACO2* was also effective along with decreased protein levels (Supplementary Fig. 1a). The analysis of publicly available gene expression datasets deposited in the Gene Expression Omnibus (GEO) highlighted that *ACO2* expression levels were reduced in human breast cancer biopsies (T) when either compared with non-tumoural adjacent counterpart (NT) or with normal breast tissue from other individuals (H) (Fig. 1b, c). The Kaplan–Meier Plotter demonstrated that the high expression of *ACO2* in human tumour specimens is associated with good clinical outcomes in terms of relapse-free survival (RFS)^26^ (Supplementary Fig. 1b–d). Less than 1% of genetic alterations were instead identified for the *ACO2* gene in breast cancer according to the TCGA data set (Supplementary Fig. 1e). Prompted by the evidence that ACO2 levels are downregulated in breast cancer cells and in human breast cancer specimens, we transiently overexpressed myc-tagged ACO2 in MCF-7 cells (Supplementary Fig. 1f), demonstrating that high levels of ACO2 reduced cell proliferation (Supplementary Fig. 1g). To corroborate this result, we generated MCF-7 cells with stable overexpression of ACO2, and we choose to perform our analyses on two independent clones (#2 and #4) with different degrees of ACO2 overexpression as well as enzymatic activity (Fig. 1d, e). Trypan blue exclusion test (Fig. 1f), bromodeoxyuridine (BrdU) incorporation (Fig. 1g; Supplementary Fig. 1h) and analysis of cytosine levels (Supplementary Fig. 1i) demonstrated dose-dependent inhibition of cell proliferation in ACO2-overexpressing clones with respect to control MCF-7 cells bearing the empty vector (EV).

### ACO2 overexpression boosts mitochondrial metabolism

**Fig. 2:**
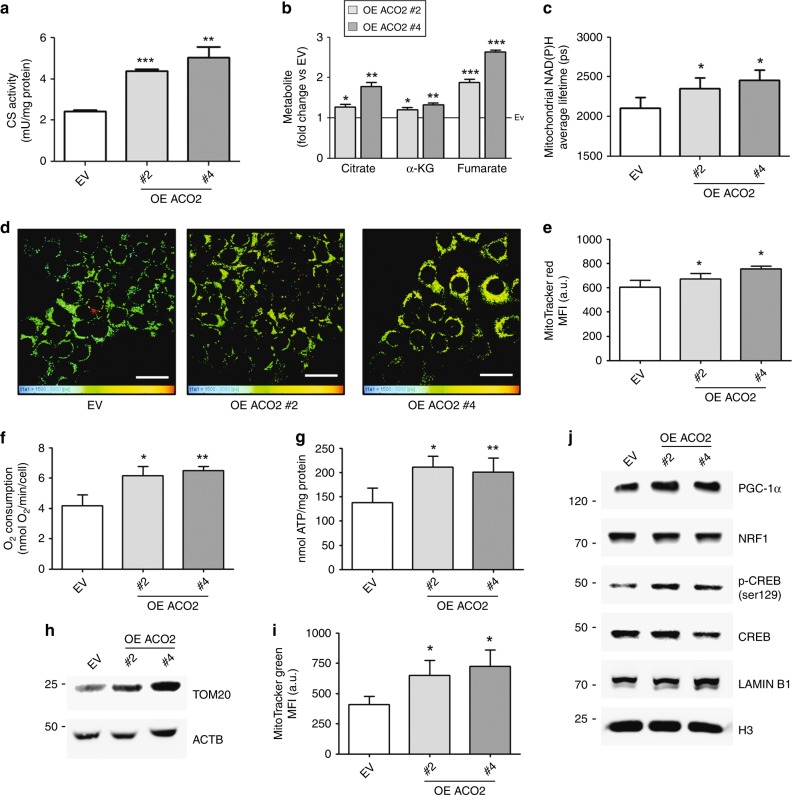
ACO2 overexpression boosts mitochondrial metabolism and biogenesis. **a** Spectrophotometric determination of citrate synthase (CS) activity (*n* = 3; **p < 0.01, ***p < 0.001 vs EV). **b** Determination of TCA cycle intermediates by colorimetric assay. Data are shown as fold change vs EV, represented by a solid line (*n* = 3; *p < 0.05, **p < 0.01, ***p < 0.001 vs EV). **c** Average mitochondrial NAD(P)H lifetime retrieved from the NAD(P)H FLIM images (*p < 0.05 vs EV). **d** Representative FLIM images where mitochondrial NAD(P)H responses were separated by the rest of the autofluorescence (cytoplasm, nuclei) by means of fluorescence intensity analysis (scale bar = 20 µm). **e** Cytofluorimetric analysis in FL-2 channel of mitochondrial membrane potential by incorporation of MitoTracker Red. MFI mean fluorescence intensity, a.u. arbitrary unit (*n* = 3; *p < 0.05 vs EV). **f** Measurement of oxygen consumption by Clark’s electrode (*n* = 3; *p < 0.05, **p < 0.01 vs EV). **g** Determination of ATP content by fluorometric assay (*n* = 3; *p < 0.05, **p < 0.01 vs EV). **h** Representative western blot (*n* = 3) showing levels of the mitochondrial protein TOM20 as a marker of mitochondrial mass in comparison with ACTB used as loading control. **i** Cytofluorimetric analysis in FL-1 channel of mitochondrial mass by incorporation of MitoTracker Green. MFI mean fluorescence intensity, a.u. arbitrary unit (*n* = 3; *p < 0.05 vs EV). **j** Representative western blot (*n* = 3) showing nuclear levels of transcription factor/co-activators involved in mitochondrial biogenesis. Histone H3 and LAMIN B1 were used as loading controls

Mitochondrial metabolism was then monitored to reveal any influence due to the stable increase of ACO2 expression in MCF-7 cells. We observed an increase in the citrate synthase (CS) activity (Fig. 2a) and consequently of the levels of the TCA cycle intermediates citrate, α-KG and fumarate in ACO2-overexpressing cells (Fig. 2b). To investigate whether increased TCA cycle flux could result in activation of ETC, we performed lifetime imaging of NADH, which is extensively used to monitor changes in metabolism.^27^ NADH has either a short or long fluorescence lifetime component depending on whether it is in a free or protein-bound state, respectively.^28^ Since metabolic states can change the extent of NADH enzymatic binding, fluorescence lifetime imaging microscopy (FLIM) permits us to investigate, with submicrometric resolution and organelle specificity, whether the induced alteration in the TCA cycle was associated with increased binding of the reduced cofactor NADH. Figure 2c shows a significant increase in the average mitochondrial NAD(P)H lifetime, retrieved from the NAD(P)H FLIM images reported in Fig. 2d, in ACO2-overexpressing cells, thus indicating an increase in the enzyme-bound state of NADH species corresponding to higher mitochondrial NADH levels used by ETC. The elevated mitochondrial membrane potential (Fig. 2e), oxygen respiration (Fig. 2f) and ATP production (Fig. 2g) corroborated that ACO2 overexpression boosts OXPHOS in MCF-7 cells in a dose-dependent fashion. To test whether these changes were associated with an increase in mitochondrial mass, we assessed the relative amount of the mitochondrial translocase TOM20 with respect to the cytosolic protein ACTB (Fig. 2h) and the fluorescence intensity of mitochondrial-selective labels (Fig. 2i; Supplementary Fig. 2a). These assays demonstrated that ACO2-overexpressing cells have an increased mitochondrial content along with expression of TCA cycle enzymes (Supplementary Fig. 2b, c) and of both mitochondrial- and nuclear-encoded genes of the OXPHOS complexes (Supplementary Fig. 2d–f). Consistently, the analysis of nuclear factors involved in mitochondrial biogenesis, including PGC-1α and ser129-phosphorylated CREB (p-CREB), were more highly detectable in ACO2-overexpressing clones (Fig. 2j). The unaltered expression of the mitochondrial chaperones HSP60 and HSP75 suggested a preserved mitochondrial homoeostasis in ACO2-overexpressing cells (Supplementary Fig. 2c).

### ACO2 overexpression affects metabolic features of cancer cells and differentiated mammary epithelial cells

**Fig. 3:**
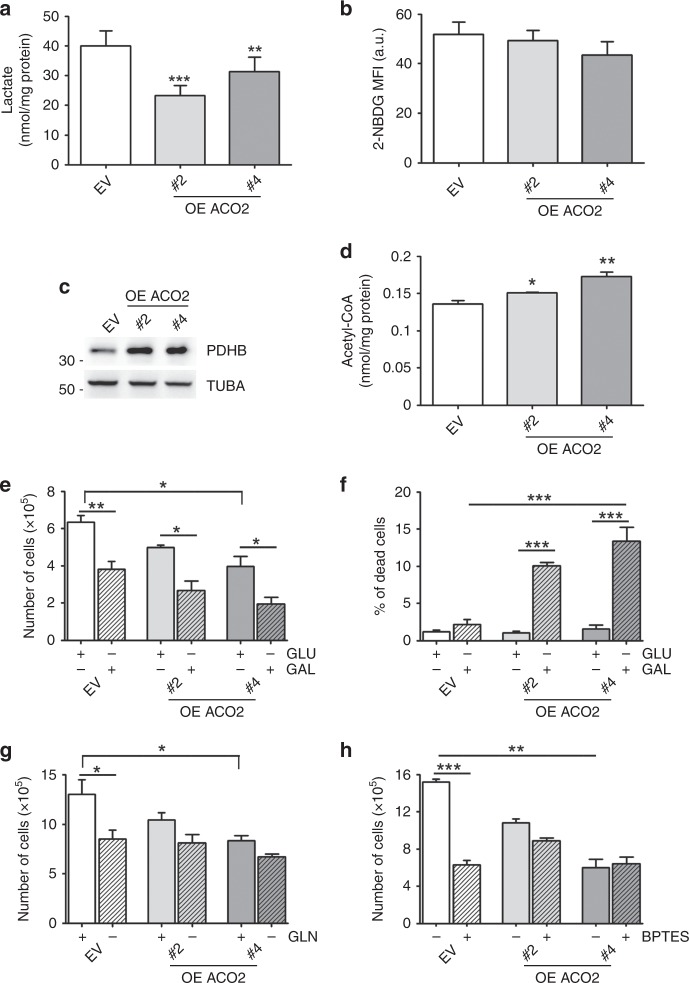
ACO2 overexpression affects aerobic glycolysis and glutamine addiction. **a** Extracellular lactate content measured by an enzymatic/spectrophotometric combined technique (*n* = 6, **p < 0.01, ***p < 0.001 vs EV). **b** Glucose uptake assessed by cytofluorimetric analysis in FL-2 channel assessed by using the fluorescent analogue of glucose 2-NBDG. MFI mean fluorescence intensity, a.u. arbitrary unit (*n* = 4). **c** Representative western blot (*n* = 3) showing the level of a subunit of the mitochondrial PDH complex. ACTB was used as loading control. **d** Determination of acetyl-CoA levels by HPLC (*n* = 3; *p < 0.05, **p < 0.01 vs EV). **e** Cell proliferation assayed by Trypan blue direct cell-counting procedure after incubation with galactose (GAL) or glucose (GLU) for 48 h (*n* = 3; *p < 0.05, ***p < 0.01 as indicated). **f** Percentage of Trypan blue-positive cells determined after incubation with galactose (GAL) or glucose (GLU) for 48 h (*n* = 3; ***p < 0.001 as indicated). **g** Cell proliferation assayed by Trypan blue direct cell-counting procedure after glutamine (GLN) depletion for 24 h (*n* = 3; *p < 0.05 as indicated). **h** Cell proliferation assayed by Trypan blue direct cell-counting procedure after glutaminase inhibitor (BPTES) treatment for 24 h (*n* = 3; **p < 0.01, ***p < 0.001 as indicated)

To assess whether improved mitochondrial metabolism promoted changes in typical features of cancer metabolism, we focused our attention on aerobic glycolysis and glutamine addiction in MCF-7 cells. After ACO2 overexpression, we observed the reduction of extracellular lactate release (Fig. 3a) that was not ascribable to altered glucose uptake, as demonstrated by the unaffected incorporation of the glucose fluorescent analogue 2-NBDG (Fig. 3b). Since this pattern was paralleled by an upregulation of the pyruvate dehydrogenase complex (i.e., β-subunit of the E1 component, PDHB) (Fig. 3c) and augmented acetyl-CoA levels (Fig. 3d), a more efficient way to funnel pyruvate towards mitochondria seemed to occur in ACO2- overexpressing cells. To demonstrate that the enhancement of oxidative metabolism is the cause of MCF-7 decreased proliferation observed after ACO2 overexpression, we replaced glucose with galactose to force cells to use OXPHOS for ATP production.^29^ In this condition, control MCF-7 cells decreased their proliferation (Fig. 3e), whereas ACO2-overexpressing cells underwent cell death (Fig. 3f).

Concerning glutamine metabolism, no alteration of glutamine and of glutamate levels was evidenced in our clones (Supplementary Fig. 3a). However, when cells were subjected to glutamine deprivation, we observed a significant decrease of cell proliferation in control cells with respect to ACO2-overexpressing cells (Fig. 3g) and no sign of apoptosis (data not shown). Treatments with the allosteric inhibitor of glutaminase, BPTES, had similar effects, thus highlighting that MCF-7 cells are less dependent on glutamine metabolism after ACO2 overexpression (Fig. 3h).

In parallel to metabolic aspects of cancer cells, we also monitored a typical metabolic trait of differentiated epithelial mammary cells, consisting of the accumulation of milk components or precursors. In fact, a decreased proliferation of breast cancer cells can be accompanied by cell differentiation, including MCF-7.^30^ The increased levels of UDP-galactose, combined with UTP decline, indicated the activation of the lactose biosynthetic pathway after ACO2 overexpression (Supplementary Fig. 3b). In support of a different utilisation of UDP galactose, we found an impairment of glucosamine pathways as shown by the reduction of UDP-GlcNAc and UDP-GalNac (Supplementary Fig. 3c). In this scenario, the increase in the human milk components taurine (Supplementary Fig. 3d) and albumin (Supplementary Fig. 3e) together with the accumulation of neutral lipids (Supplementary Fig. 3f) demonstrated that ACO2-overexpressing clones have a more differentiated phenotype.

### ACO2 overexpression induces oxidative stress-mediated autophagic/mitophagic flux

**Fig. 4:**
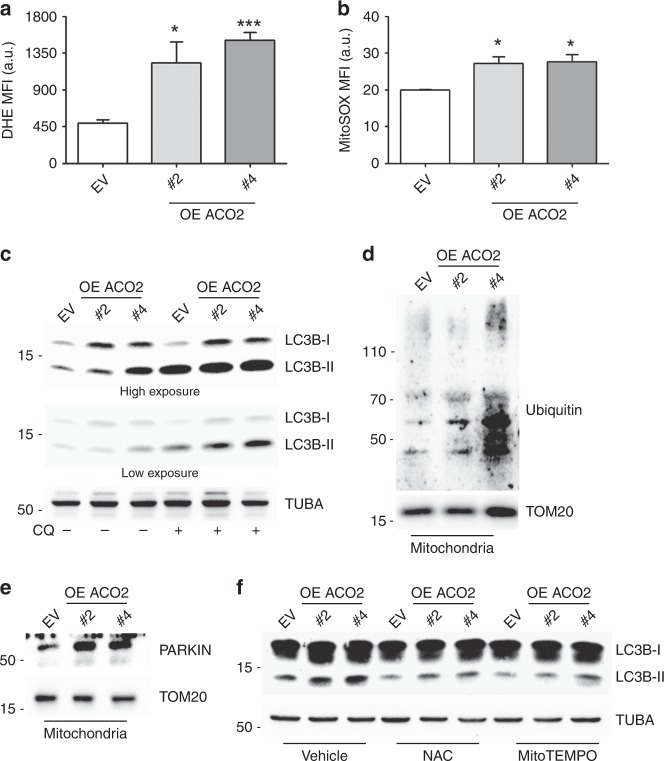
ACO2 overexpression enhances ROS production and autophagic/mitophagic response. **a** Determination of ROS levels by cytofluorimetric analysis in the FL-2 channel assessed by using the probe Dihydroethidium (DHE). MFI mean fluorescence intensity, a.u. arbitrary unit (*n* = 3; *p < 0.05, ***p < 0.001 vs EV). **b** Determination of mitochondrial ROS levels by cytofluorimetric analysis in the FL-2 channel assessed by using the probe MitoSOX. MFI mean fluorescence intensity, a.u. arbitrary unit (*n* = 3; *p < 0.05 vs EV). **c** Representative western blot (*n* = 3) analysis of the autophagy marker LC3B-II with/without the autophagy inhibitor chloroquine (CQ). α-TUBULIN (TUBA) was used as loading control. **d** Representative Western blot (*n* = 4) analysis of ubiquitinated proteins in the mitochondrial fraction. TOM20 was used as loading control. **e** Representative western blot (*n* = 3) analysis of PARKIN recruitment to mitochondrial fraction. TOM20 was used as loading control. **f** Representative western blot (*n* = 3) analysis of LC3B protein with/without 24 h of treatment with the antioxidants N-acetylcysteine (NAC) and MitoTEMPO. TUBA was used as loading control

The enhancement of mitochondrial metabolism in ACO2-overexpressing cells may be associated with augmented oxidative stress. Consistently, we observed an increase in intracellular (Fig. 4a) and mitochondrial ROS (Fig. 4b), as well as carbonylated proteins (Supplementary Fig. 4a) after ACO2 overexpression. This pattern was accompanied by variations in the antioxidant system as demonstrated by the upregulation of *GCLC* and *SOD2* genes (Supplementary Fig. 4b), by decreased levels of reduced glutathione (Supplementary Fig. 4c) and by increased NADP^+^/NADPH ratio (Supplementary Fig. 4d). No sign of nitrosative stress was instead identifiable as suggested by the unchanged levels of intracellular nitrite and nitrate (Supplementary Fig. 4e, f). We then tested the functional role of oxidative stress in cancer-related processes by incubating MCF-7 cells with the ROS scavenger N-acetylcysteine (NAC). We observed that ROS abrogation is associated with increased cell growth and with molecular features of epithelial–mesenchymal transition phenotype (i.e. upregulation of *VIM* and downregulation of *CDH1*) in ACO2-overexpressing cells (Supplementary Fig. 4g, h).

ROS production by mitochondria is known to promote autophagy, which is necessary for the removal of damaged/exhausted mitochondria via mitophagy.^31^ Therefore, we measured the levels of lipidated LC3B protein (LC3B-II) as a marker of autophagy activation. The increased levels of LC3B-II in our clones and the further accumulation observed in the presence of the autophagy inhibitor chloroquine (CQ) demonstrated that ACO2 overexpression triggers an active autophagic flux (Fig. 4c). We further revealed an increase in mitophagy, as shown by the high levels of ubiquitinated proteins (Fig. 4d) and of PARKIN recruitment (Fig. 4e) in mitochondrial fractions. These results were corroborated by the co-localisation of mitochondrial matrix-targeted mitoDsRed with GFP-LC3 (Supplementary Fig. 5a) and by the high expression of the mitochondria fission protein DRP1^32^ in ACO2 clones (Supplementary Fig. 5b). We definitely demonstrated that autophagic induction in ACO2-overexpressing cells was a consequence of ROS production by the treatment with the ROS scavengers NAC, MitoTEMPO and ascorbate that were able to inhibit the accumulation of LC3B-II protein (Fig. 4f; Supplementary Fig. 5c). To define the relevance of autophagy induction in our cells, we inhibited it by preventing the acidification of lysosomes with bafilomycin A1 (BafA1). The reduction in the number of ACO2-overexpressing cells (Supplementary Fig. 5d) and the appearance of the cleaved fragment of PARP-1 protein, a typical marker of apoptosis, after BafA1 (Supplementary Fig. 5e) suggested an important contribution of autophagy/mitophagy in the maintenance of cell homoeostasis buffering ROS.

### Autophagy induction downstream of ACO2 overexpression is associated with ROS/FoxO1 signalling

**Fig. 5:**
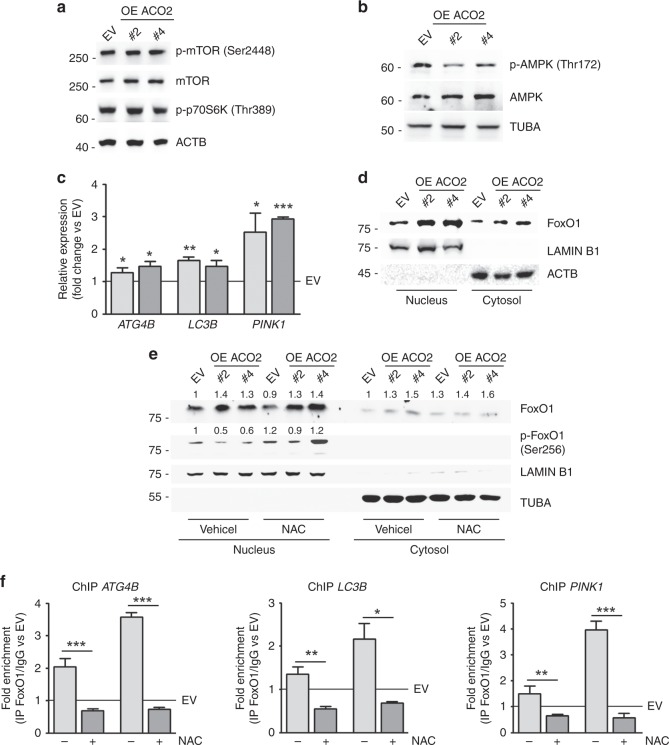
ACO2 overexpression promotes autophagy via ROS/FoxO1 signalling. **a** Representative Western blot (*n* = 3) analysis of activated/phosphorylated enzymes of the mTOR pathway. ACTB was used as loading control. **b** Representative Western blot (*n* = 3) analysis of active Thr172-phosphorylated AMPK (p-AMPK). TUBA was used as loading control. **c** RT-qPCR analysis of autophagic genes by using β-actin as reference control. Data are shown as fold change vs EV, represented by a solid line (*n* = 3; *p < 0.05, ** *p* < 0.01, ***p < 0.001 vs EV). **d** Representative western blot (*n* = 3) analysis of FoxO1 localisation in nuclear and cytosolic fractions by using LAMIN B1 and ACTB as loading and purity controls. **e** Representative western blot (*n* = 3) analysis of FoxO1 and Ser256-phosphorylated FoxO1 (p-FoxO1) in nuclear and cytosolic fractions with/without N-acetylcysteine (NAC). LAMIN B1 and TUBA were used as loading and purity controls. Numbers refer to densitometric analyses of FoxO1 and p-FoxO1/FoxO1 after normalisation on LAMIN B1 or TUBA and are expressed as fold change vs EV. **f** Chromatin immunoprecipitation assay (ChIP) performed by using anti-FoxO1 antibody in nuclear fractions obtained after treatment with NAC treatment for 24 h. Data are expressed as fold enrichment of IP FoxO1/ IgG vs EV, represented by a solid line (*n* = 3; *p < 0.05, **p < 0.01, ***p < 0.001)

To identify the molecular pathways behind autophagic response in ACO2-overexpressing cells, we firstly focused on the key autophagy inhibitor mTOR.^33^ The unchanged levels of Ser2448-phosphorylated mTOR and its target Thr389-phosphorylated p70S6 kinase excluded nutrient-limiting conditions as determinants of ROS-mediated autophagic flux in our clones (Fig. 5a). Such evidence is corroborated by the low levels of Thr172 phosphorylation of the energy sensor AMPK (Fig. 5b). In support of this, we showed that our cells retain the ability to trigger autophagy when challenged with amino acid starvation (achieved by EBSS medium incubation), which is a canonical stimulus impinging on mTOR activity (Supplementary Fig. 5f). Then, we demonstrated that the activation of autophagic flux was associated with transcriptional upregulation of the key autophagic genes *LC3B* and *ATG4B* as well as of the mitophagic gene *PINK1* (Fig. 5c). Considering that all autophagic genes here analysed are targets of FoxO proteins and that we have previously demonstrated that FoxO1 is a key transcription factor known to connect metabolism-associated oxidative stress with autophagy/mitophagy induction,^24^ we assessed the levels of FoxO1 in our experimental conditions. We firstly revealed that FoxO1 is highly abundant in the nuclear fractions of ACO2-overexpressing cells with respect to those of control (Fig. 5d). To determine whether FoxO1 nuclear localisation was a consequence of ROS buildup, we performed NAC treatment, but we did not evidence any specific change in its subcellular distribution (Fig. 5e). We thus analysed the levels of Ser256-phosphorylated FoxO1 demonstrating that this modification is less marked in nuclear fractions of untreated ACO2-overexpressing cells while it increases after NAC treatment (Fig. 5e). To evidence whether this scenario was associated with changes in FoxO1 recruitment at autophagic gene promoters, we tested sequences known to be targeted by FoxO1 performing ChIP analysis after NAC treatment. This experiment demonstrated that FoxO1 is highly enriched at *LC3B*, *ATG4B* and *PINK1* promoters (Fig. 5f) in untreated ACO2-overexpressing cells, whereas its binding is largely disrupted after ROS removal by NAC.

## Discussion

The specialised metabolic landscape that is established in each cancer cell derives from the activation of oncogenic pathways or abrogation of tumour-suppressor signalling and is continuously shaped by the interaction with tumour microenvironment comprising stromal/immune cells and nutrient/oxygen availability.^3^ In some cases, cancer metabolic reprogramming can be a direct consequence of oncogenic driver mutations affecting metabolic genes as shown for half of the enzymes belonging to the TCA cycle: fumarate hydratase, succinate dehydrogenase, isocitrate dehydrogenase 2 and malate dehydrogenase 2.^1,2^ All these mutations are responsible for cellular transformation due to accumulation of TCA cycle intermediates or aberrant production of oncometabolites. Changes in the expression levels of several TCA cycle enzymes, such as CS and IDH3α,^34,35^ have also been shown to affect tumour phenotype suggesting that alteration of any step of TCA cycle can be detrimental for cell metabolic homoeostasis. Although no mutation in *ACO2* sequence has been associated with tumour susceptibility, in this paper, we have demonstrated that expression levels of ACO2 are deregulated in breast cancer and that its overexpression in MCF-7 cell line is able to dampen cell proliferation. On the contrary, the modulation of the cytosolic ACO1 was shown to have no impact on breast cancer proliferation.^36^ Besides our present work, a direct contribution of ACO2 in tumorigenesis was exclusively demonstrated in prostate cancer, and this is mainly justified by the importance of citrate metabolism in non-malignant prostate epithelial cells. In fact, prostate tissue physiologically accumulates high amount of citrate, thanks to a zinc-mediated inhibition of ACO2.^37,38^ Even though it is well known that ACO2 is not a rate-limiting enzyme of the TCA cycle, our data pointed out the importance of its reaction in promoting cancer metabolic rewiring. In fact, we demonstrated that increased levels of ACO2 in MCF-7 cells are able to affect aerobic glycolytic rate as demonstrated by the reduction of extracellular lactate efflux. This evidence could give effort to the MCF-7 glycolytic shift observed under defective assembly of iron–sulfur clusters, which are essential for aconitase activity.^39^ The unaffected glucose assimilation along with increased level of acetyl-CoA and PDH complex demonstrated a different fate for pyruvate, prevalently directed to mitochondria, in cells with increased ACO2 levels. The same changes were observed during treatment of colorectal cancer cells with dichloroacetate (DCA). This compound induces cell growth arrest by inhibiting pyruvate dehydrogenase kinase (PDK) with consequent activation of PDH, thus promoting the entry of pyruvate into the TCA cycle.^40^ We also demonstrated that ACO2 expression affects glutamine addiction of MCF-7 cells, indicating that feeding TCA cycle with pyruvate and channelling it for aerobic oxidation in the presence of adequate ACO2 levels dislodge cancer cells from using glutamine for anaplerosis.

The re-routing of glucose-derived pyruvate towards mitochondria can be considered as the guiding force involved in the proliferation inhibition of ACO2-overexpressing cells. Consistently, we observed a decrease of cell proliferation in control cells forced to use OXPHOS by galactose treatment.^29^ The enhancement of mitochondrial metabolism and the reduction of the Warburg effect also underlie the anti-proliferative effects observed after alanine aminotransferase inhibition,^41^ improved triacylglycerol catabolism in hepatocellular carcinoma^11^ and DCA treatment in colorectal cancer.^40^ Moreover, the increase in mitochondrial biogenesis due to PGC-1α activation was shown to be another efficient strategy against the Warburg effect.^42–44^ It has to be noticed that after ACO2 overexpression, we also observed an increase in mitochondrial mass associated with high levels of PGC-1α and Ser129-phosphorylated CREB. This would imply that ACO2-mediated metabolism is able to trigger a mitonuclear retrograde response culminating in mitochondrial biogenesis that needs further investigations. Whatever the causes, pyruvate redirection, mitochondrial biogenesis or both, our data support that ACO2 can boost OXPHOS weakening the Warburg effect.

Mitochondria are the major physiological source of ROS and can cause an increase in oxidative stress following enhanced metabolic rate or electron leakage to oxygen when they are dysfunctional.^45,46^ The contribution of ROS in breast cancer aetiology and progression is dual when they act as signal molecules in metabolic adaptations and proliferation pathways, as well as when they act as harmful factors, eliciting pro-apoptotic or mutagenic effects by damaging macromolecules, primarily nucleic acids.^47^ In general, low concentrations of ROS are believed to promote cancer cell survival as demonstrated for the maintenance of breast cancer stem cells and resistance to radiotherapy.^48^ High concentrations of ROS instead can promote oxidative damage and eradication of tumour cells via programmed cell death, a mechanism frequently underpinning the action of chemotherapeutic drugs. Along with dosage, also type, duration and site of generation dictate ROS functional outcome.^47^ In the context of ACO2-overexpressing cells, a deleterious effect of ROS can be envisaged after the enhancement of mitochondrial oxidative metabolism by galactose administration that commits them to cell death. Under standard glucose conditions, the viability of ACO2-overexpressing cells was assured by the induction of protective antioxidant systems and autophagy as survival mechanisms. This result is in agreement with ROS-mediated activation of autophagy occurring after DCA-dependent reorientation of pyruvate towards the TCA cycle.^40^ The role of autophagy in breast cancer as much as in the majority of tumour types is complex for the deep integration into metabolism, stress response and cell-death pathways.^49,50^ The loss of the essential autophagic protein Beclin1 in mammary epithelial cells induces tumorigenesis as a consequence of genome instability.^51^ Another tumour-suppressor role of autophagy in breast cancer entails the removal of damaged mitochondria as exemplified by the augmented proliferation and aggressiveness resulting from the downregulation of PARKIN or BNIP3, two key pro-mitophagic proteins.^52–54^ Nevertheless, autophagy induction promotes cell survival against stressful conditions (e.g. nutrient limitations, hypoxia) as described for dormant breast cancer cells responsible for tumour recurrence^50,55^ or against chemotherapeutic interventions contributing to apoptosis resistance.^56,57^ In the absence of any causal change in the nutrient-sensing pathways of mTOR and AMPK, the recruitment of the ubiquitin E3 ligase PARKIN at mitochondria with consequent increase in ubiquitin-conjugated proteins indicated that ACO2 overexpression leads to the activation of a mitophagic process. This result is also supported by the increased levels of DRP1 protein, which promotes mitochondrial fission, a mechanism generally coupled to mitophagy following oxidative stress.^58^ The fact that mitophagy occurs in parallel with mitochondrial biogenesis suggests an active turnover of mitochondria rather than a definitive disposal.^32^ Therefore, the activation of mitophagy caused by ACO2 overexpression likely accounts for the removal of damaged/exhausted mitochondria due to the enhanced oxidative metabolism in the context of a mitochondrial quality control process. It is interesting to notice that many other papers have demonstrated how modulation of autophagic/mitophagic process impacts on cancer metabolism. In particular, the Warburg effect was shown to be promoted by loss of the pro-mitophagic proteins PINK1 and PARKIN,^59–61^ while it was dampened after autophagy stimulation achieved by mTOR inhibition, serum/amino acid starvation or ATG7 overexpression.^62,63^

The transcriptional activation of *ATG4B*, *LC3B* and *PINK1* in ACO2-overexpressing cells is consistent with a lasting autophagic/mitophagic process necessary for the adaptation of cells to stable ACO2 overexpression. In our context, ROS seem to act also as signalling molecules activating the redox-sensitive transcription factor FoxO1. In fact, FoxO1 is able to couple oxidative stress to autophagic response^24,64,65^ and to participate in the retrograde response triggered by ROS following starvation or mitochondrial dysfunction.^66–68^ The molecular mechanism driving FoxO1 action upon oxidative stress mainly consists of its post-translational modification and nuclear accumulation.^69–71^ Although we observed no evident change in nuclear levels of FoxO1, the high level of FoxO1 Ser256 phosphorylation after NAC is noteworthy because this modification is present in the DNA-binding domain restraining FoxO1 transcriptional activity.^72^ Consistently, we observed a reduced FoxO1 occupancy at autophagic/mitophagic promoters after ROS abrogation. Therefore, FoxO1 may be a key player in the communication between mitochondria and the nucleus in cancer cells for the maintenance of oxidative metabolism through mitochondrial quality control. Ongoing research is devoted to the evaluation of other redox-sensitive transcription factors in the metabolic adaptation induced by ACO2 overexpression.

Overall, this work has evidenced that ACO2 expression modifies metabolic features of MCF-7 cells imposing repression of the Warburg effect and repurposing pyruvate in the aerobic route speeding OXPHOS. This scenario is accompanied by a decrease in cell proliferation associated with ROS/FoxO1 signalling necessary for preserving cellular homoeostasis. These data suggest that ACO2 can be included in metabolic reprogramming exploited by cancer cells, considering that it can be found deregulated in breast, gastric and prostate tumour biopsies.^16,37^ Future studies are necessary to enlarge this evidence on other cell systems or in vivo models with the final aim to evaluate whether ACO2 can be a suitable therapeutic target or a biomarker of metabolic vulnerability. In fact, it is worth to highlight that this neglected enzyme of the TCA cycle seems to interfere with different aspects of cancer metabolism from aerobic glycolysis to glutamine addiction and autophagy, without excluding the relevant implication it may have in cancer immunometabolism by supplying *cis*-aconitate for the production of the anti-inflammatory metabolite itaconate.^73^

## Supplementary information

Supplemental Material

## Data Availability

All data and materials generated in this study are available from the corresponding author.
