# Supplementary material for: Aconitase 2 inhibits the proliferation of MCF-7 cells promoting mitochondrial oxidative metabolism and ROS/FoxO1-mediated autophagic response
Source: Br J Cancer. 2019 Dec 10;122(2):182–93. doi: 10.1038/s41416-019-0641-0 (PMC7051954; doi:10.1038/s41416-019-0641-0)
Supplement: Supplementary file 1 — Supplemental Material [file 41416_2019_641_MOESM1_ESM.docx]

**SUPPLEMENTARY MATERIALS**

Content:

Supplementary Tables 1 and 2

Supplementary Figures 1-5 and Legends

**Supplementary Table 1:** Primers used for gene expression analysis

| Gene | Forward Primer | Reverse Primer |
| --- | --- | --- |
| *CS* | TCCGACCCTTACCTGTCCTT | ACTTCCTGATTTGCCAGTCC |
| *ACO2* | ACAGCCTACTGGTGACTCGG | GGGCTCAAAGTGGCTCATC |
| *IDH2* | CCATCATCTGCAAAAACATCC | CCAATGGTGATGGGCTTG |
| *SUCGL2* | AGCCAGCCAACTTCTTGGA | GGATGGCTTCAACCTTAGGA |
| *SDH-A* | TGGGAACAAGAGGGCATCTG | CCACCACTGCATCAAATTCAT |
| *FH* | TGAATGTTTTCAAGCCAATGAT | CCACCACGCAGTTTTCTGTA |
| *MDH2* | CAGGACCAGCTGACAGCAC | AGCCTGCTCCGGCTTTAG |
| *IDH3B* | GCCCAATCTCTATGGGAACA | CAGGGACCACACCAGCTC |
| *MT-ND1* | CTACTACAACCCTTCGCTGAC | GGATTGAGTAAACGGCTAGGC |
| *MT-ND4* | CTAGGCTCACTAAACATTCTA | CCTAGTTTTAAGAGTACTGCG |
| *MT-ND6* | CTAAAACACTCACCAAGACC | GGAATGATGGTTGTCTTTGG |
| *MT-CYB* | TGAAACTTCGGCTCACTCCT | AATGTATGGGATGGCGGATA |
| *MT-CO1* | GATTTTTCGGTCACCCTGAAG | CTCAGACCATACCTATGTATC |
| *MT-CO2* | CTATCCTGCCCGCCATCATC | GATTAGTCCGCCGTAGTCGG |
| *MT-CO3* | CACATCCGTATTACTCGCATC | GAAGTACTCTGAGGCTTGTAG |
| *MT-ATP6* | CACACCTACACCCCTTATCCC | TCATTATGTGTTGTCGTGCAG |
| *MT-ATP8* | ATGGCCCACCATAATTACCC | GCAATGAATGAAGCGAACAG |
| *NDUFB6* | TGGTCCATGGGGTATACAAAAAGA | TCTCCAGAATTGTATCACCAGGG |
| *NDUFV1* | GCGGGTATCTGTGCGTTTC | GAACCTTTCAGCCTCCAGTCA |
| *CYC1* | TACGGACACCTCAGGCAGT | CACGGTGAGACCACGGATAG |
| *UQCRC1* | GGATATGGCCCCATTGAGCA | CCGAAGTGCTGTGTTTGTGG |
| *COX4I1* | CCCGGCATTTTACGACGTTC | CAACATTCTGCCGCCACTG |
| *COX5A* | GGGCATGCAGACGGTTAAATG | AAGCCCATCCATGCGGTTTA |
| *ATP50* | CGCGTTTCTCTCTTCCCACT | TACCTGAACAGGAGGCCTCA |
| *GCLC* | AGGAGCGAGGACTGGAGCCAT | GCAACATGCTGGGCCAGGAGA |
| *SOD2* | GCAAGGAACAACAGGCCTTA | AAGAGCTTAACATACTCAGCATAAC |
| *ATG4B* | CCATCGTGTGGGGTTTTTC | AGAATCTAGGGACAGGTTCAGGA |
| *LC3B* | TGCGGGCTGAGGAGATACAA | TACTCTTTGTTCGAAGGTGCGG |
| *PINK1* | AGTCCATTGGTAAGGGCTGC | GAACCTGCCGAGATGTTCCA |
| *CDH1* | CGTCCTGGGCAGAGTGAAT | ACACCATCTGTGCCCACTTT |
| *VIM* | CGGGAGAAATTGCAGGAGGA | AAGGTCAAGACGTGCCAGAG |

**Supplementary Table 2:** Primesr used for ChIP analysis

| Gene | Forward Primer | Reverse Primer |
| --- | --- | --- |
| *ATG4B* | TGGGTGACAGAGTGGGACT | GTATCTTCCTGCGCTGGTTG |
| *LC3B* | CAGCCCTCCCGGATACTG | GCCGAGATTGTACTACTGCC |
| *PINK1* | GCCAGTACCAGCATAGCG | CCCGCCGGTCACAACAAA |

**Supplementary Figures**

**Supplementary Figure 1**


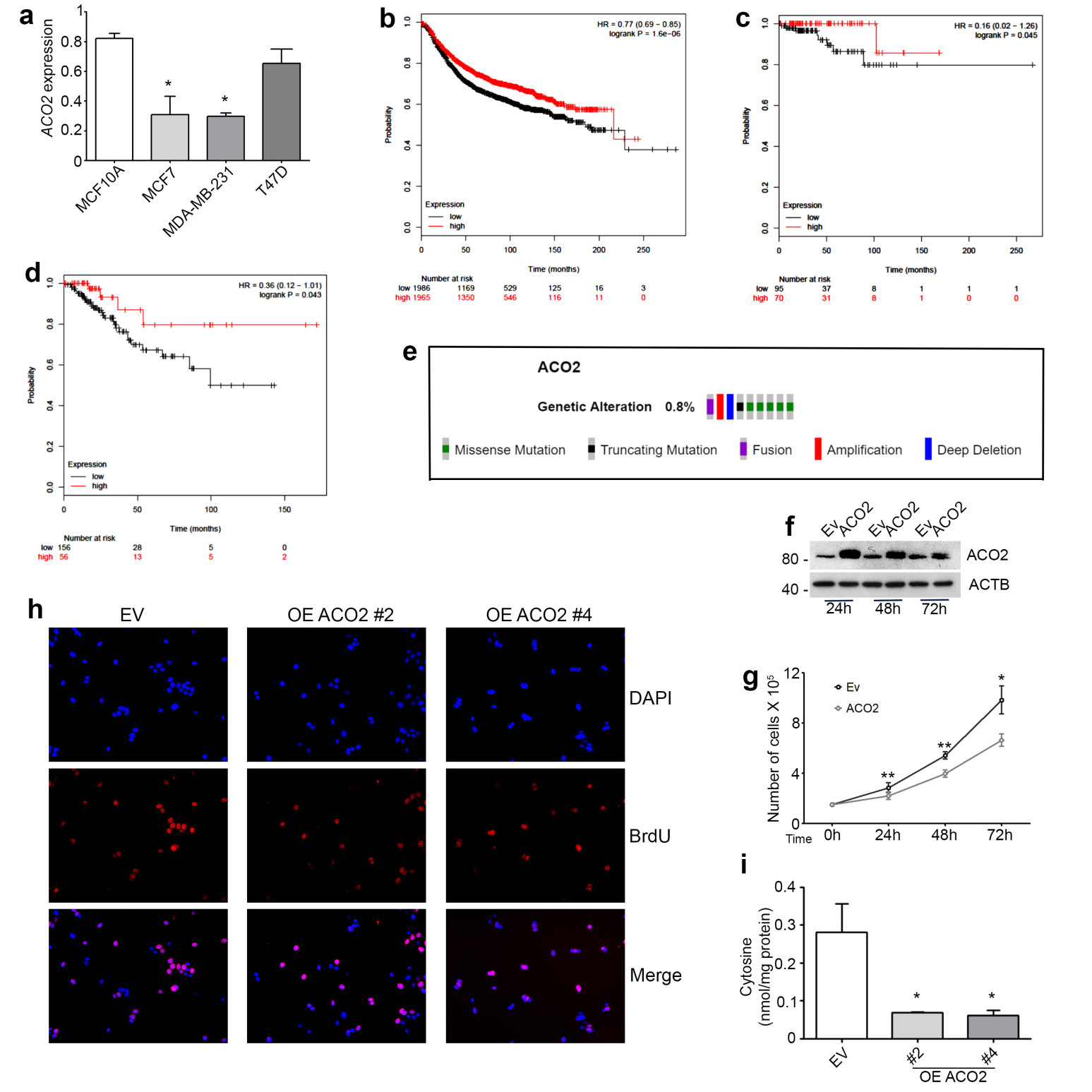


**Supplementary Figure 2**


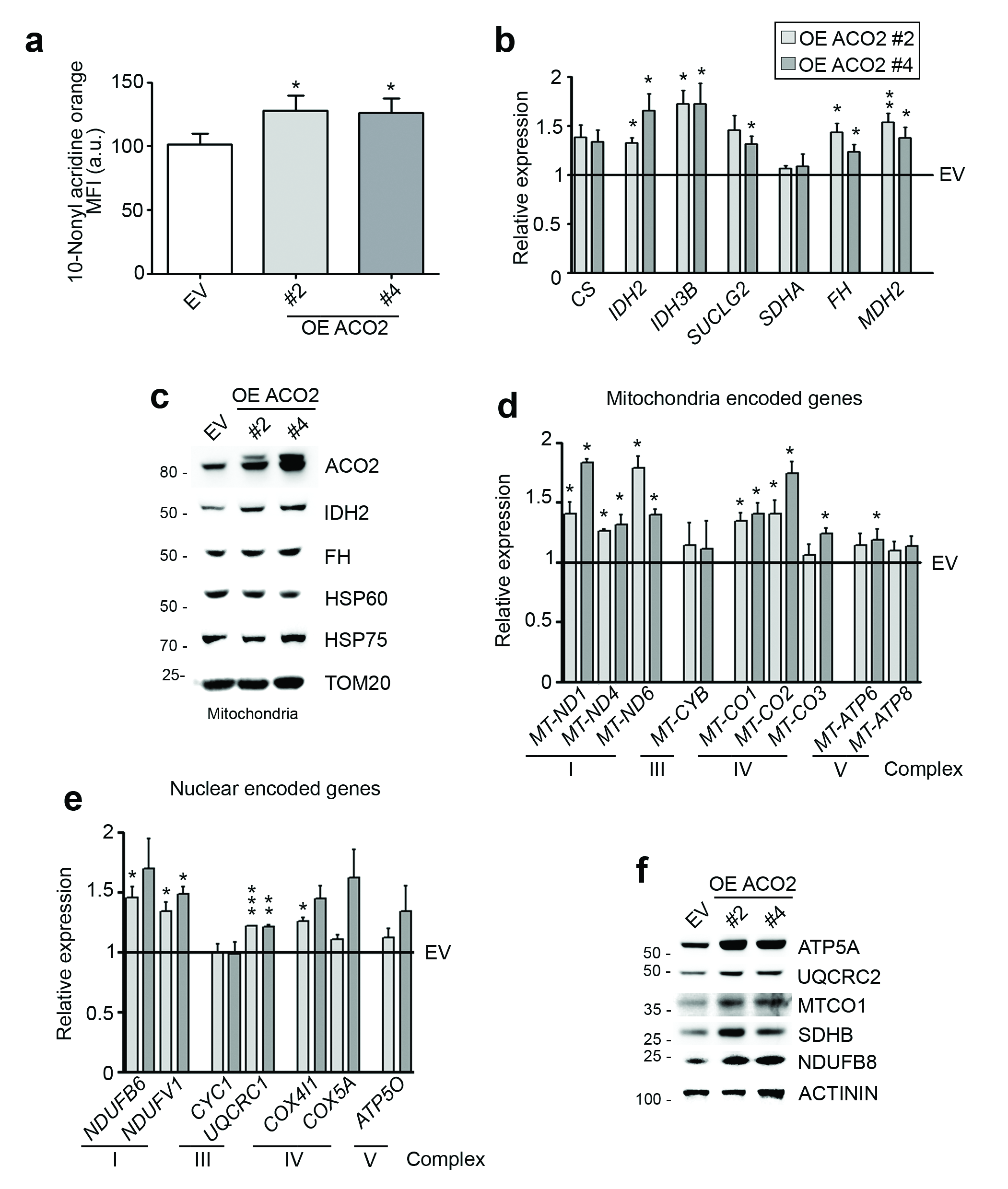


**Supplementary Figure 3**

**
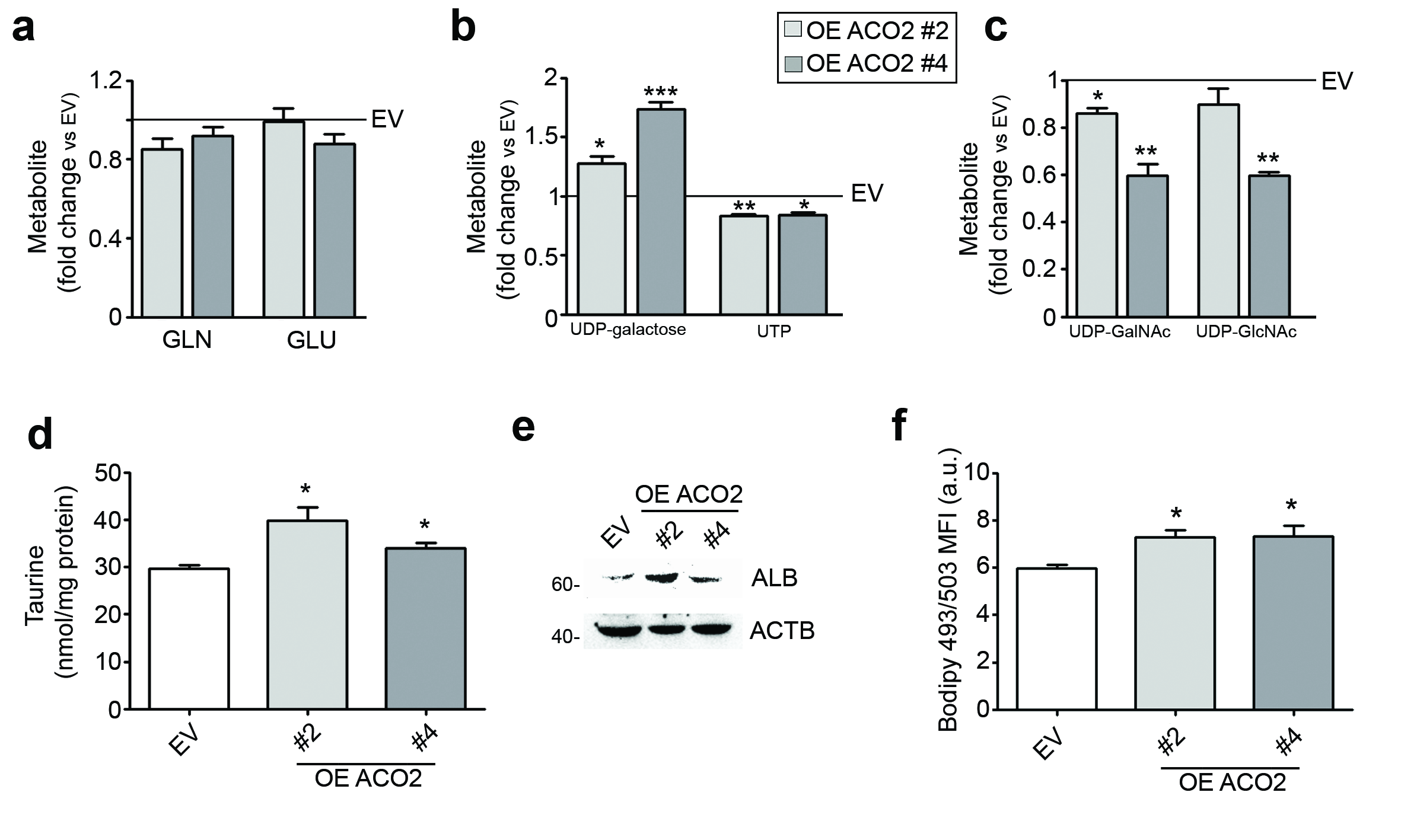
**

**Supplementary Figure 4
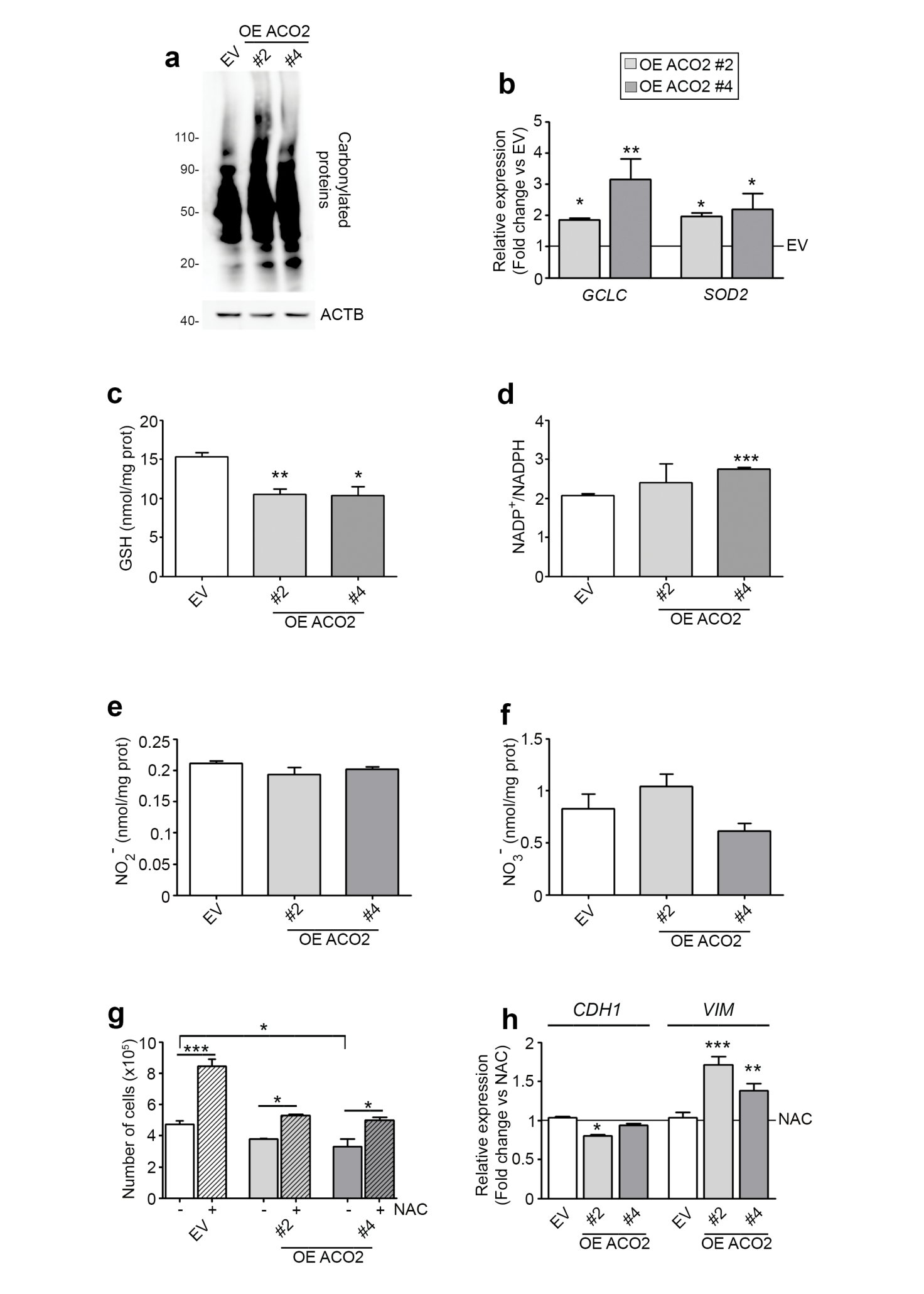
**

**Supplementary Figure 5**


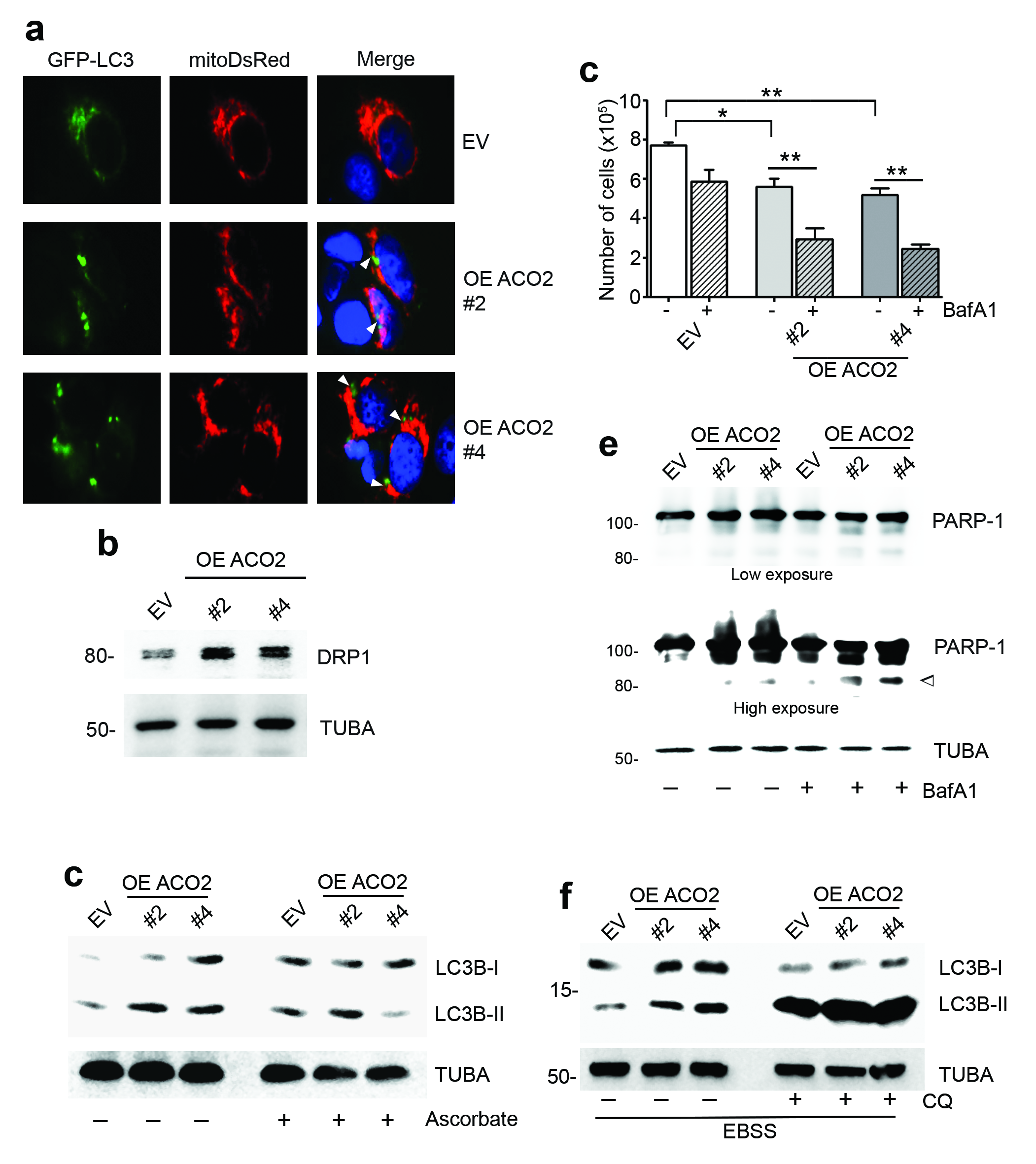


**Supplementary Figure Legends**

**Supplementary Figure 1. ACO2 overexpression inhibits MCF-7 cell proliferation.**

**a)** RT-qPCR analysis of *ACO2* gene in several cell lines using β-Actin as reference control (n=3; * *p<0.05, vs* MCF10A); **b)** Relapse-free survival (RFS) according to ACO2 expression in breast cancer RNA-seq analysis with no distinction in the stage of disease (n=2951); **c)** RFS according to ACO2 expression in breast cancer RNA-seq analysis at stage 1 of disease (n=165); **d)** RFS according to ACO2 expression in breast cancer RNA-seq analysis at stage 3 of disease (n=212); **e)** Mutational analysis of *ACO2* gene in breast cancer according to TGCA database; **f)** Representative Western blot (n=3) analysis showing ACO2 protein levels after transient overexpression of MCF-7 cells for 24, 48 and 72 hours (h). ACTB was used as loading control; **g)** Cell proliferation assayed by Trypan blue direct cell counting procedure after transient overexpression of MCF-7 cells for 24, 48 and 72 hours (h). (n=3; * *p<0.05,* ** *p<0.01 vs* EV); **h)** Representative immunofluorescence images of BrdU incorporation assay in EV- and ACO2-overexpressing clones; **1)** Determination of cytosine levels performed by HPLC analysis (n=3; * *p<0.05, vs* EV).

**Supplementary Figure 2. ACO2 overexpression increases TCA cycle and oxidative phosphorylation mitochondrial equipment.**

**a)** Cytofluorimetric analysis of mitochondrial mass by incorporation of 10-Nonyl acridine orange. MFI: mean fluorescence intensity. a.u.: arbitrary unit. (n=3; * *p<0.05, vs* EV); **b)** RT-qPCR analysis of TCA cycle genes using β-Actin as a reference control. Data are shown as fold change *vs* EV, represented by a solid line (n=3; * *p<0.05,* ** *p<0.01, vs* EV); **c)** Representative Western blot (n=3) analysis of some TCA cycle enzyme and chaperones in mitochondrial fraction. TOM20 was used as loading control; **d)** RT-qPCR analysis of mitochondrial encoded genes for oxidative phosphorylation complexes. β-Actin was used as reference control. Data are shown as fold change *vs* EV, represented by a solid line (n=3; * *p<0.05,* *vs* EV); **e)** RT-qPCR analysis of nuclear-encoded genes for oxidative phosphorylation complexes. β-Actin was used as reference control. Data are shown as fold change *vs* EV, represented by a solid line (n=3; * *p<0.05,* ** *p<0.01,* *** *p<0.001, vs* EV); **f)** Representative Western blot (n=3) analysis of subunits of mitochondrial complexes. ACTININ was used as loading control.

**Supplementary Figure 3. ACO2 overexpression increases the metabolic traits of differentiated breast epithelial cells.**

**a)** Determination of glutamine (GLN) and glutamate (GLU) levels performed by HPLC analysis; Data are shown as fold change *vs* EV, represented by a solid line (n=3); **b)** Determination of metabolite involved in lactose biosynthesis performed by HPLC analysis; Data are shown as fold change *vs* EV, represented by a solid line (n=3; * *p<0.05,* ** *p<0.01,* *** *p<0.001, vs* EV); **c)** Determination of uridine-diphosphate-n-acetylgalactosamine (UDP-GalNAc) and Uridine diphosphate-N-acetylglucosamine (UDP-GlcNAc) levels performed by HPLC analysis; Data are shown as fold change *vs* EV, represented by a solid line (n=3; * *p<0.05,* ** *p<0.01, vs* EV); **d)** Determination of Taurine levels performed by HPLC analysis (n=3; * *p<0.05,* *vs* EV); **e)** Representative Western blot (n=3) analysis of Albumin (ALB) levels using ACTB as loading control; **f)** Cytofluorimetric analysis of neutral lipid content by incorporation of Bodypy 493/503. MFI: mean fluorescence intensity. a.u.: arbitrary unit. (n=3; * *p<0.05, vs* EV).

**Supplementary Figure 4. ACO2 overexpression induces oxidative stress.**

**a)** Representative Western blot (n=3) analysis of oxidative stress damage to proteins after derivatization of carbonyl groups. ACTB was used as loading control; **b)** RT-qPCR analysis of antioxidant genes using β-Actin as reference control; D

ata are shown as fold change *vs* EV, represented by a solid line (n=3; * *p<0.05,* ** *p<0.01, vs* EV); **c)** Determination of GSH levels performed by HPLC analysis (n=3; * *p<0.05,* ** *p<0.01, vs* EV); **d)** Determination of NADP^+^/NADPH ratio performed by HPLC analysis (n=3; *** *p<0.001, vs* EV); **e)** Determination of NO_2_^-^ levels performed by HPLC analysis (n=3); **f)** Determination of NO_3_^-^ levels performed by HPLC analysis (n=3); **g)** Cell proliferation assayed by Trypan blue direct cell counting procedure after N-acetylcysteine (NAC) treatment for 24 hours (n=4; * *p<0.05,* *** *p<0.001* as indicated); **h)** RT-qPCR analysis of genes involved in epithelial to mesenchymal transition using β-Actin as reference control; Data are shown as fold change *vs* NAC-treated cells, represented by a solid line (n=3; * *p<0.05,* ** *p<0.01,* *** *p<0.001 vs* NAC).

**Supplementary Figure 5. ACO2 overexpression induces autophagy/mitophagy.**

**a)** Immunofluorescence analysis demonstrating GFP-LC3 and mitoDsRed contacts (arrowheads in merge panel) as indicators of mitophagy; **b)** Representative Western blot (n=3) analysis of DRP1 levels, a marker of mitochondrial fission. TUBA was used as loading control; **c)** Representative Western blot (n=3) analysis of LC3B levels after ascorbate treatment fro 24 hours. TUBA was used as a loading control. **d)** Cell proliferation assayed by Trypan blue direct cell counting procedure after bafilomycin A1 (BafA1) treatment for 24 hours (n=4; * *p<0.05,* ** *p<0.01,* as indicated); **e)** Representative Western blot (n=3) analysis of PARP-1 after bafilomycin A1 (BafA1) treatment for 24 hours. Arrowhead indicates the cleaved fragment of PARP-1. TUBA was used as loading control; **f)** Representative Western blot (n=3) analysis of LC3B levels after amino acid depletion obtained by incubation with Earle's Balanced Salt Solution (EBSS) medium with/without Chloroquine (CQ). TUBA was used as a loading control.
